# Comparative Proteome Analysis of Brown Adipose Tissue in Obese C57BL/6J Mice Using iTRAQ-Coupled 2D LC-MS/MS

**DOI:** 10.1371/journal.pone.0119350

**Published:** 2015-03-06

**Authors:** Juan Li, Wei-Gang Zhao, Zhu-Fang Shen, Tao Yuan, Shuai-Nan Liu, Quan Liu, Yong Fu, Wei Sun

**Affiliations:** 1 Department of Endocrinology, Key Laboratory of Endocrinology of Ministry of Health, Peking Union Medical College Hospital, Chinese Academy of Medical Science and Peking Union Medical College, Beijing, China; 2 State Key Laboratory of Bioactive Substances and Functions of Natural Medicines, Institute of Materia Medica, Chinese Academy of Medical Sciences and Peking Union Medical College, Diabetes Research Center of Chinese Academy of Medical Sciences, Beijing, China; 3 Core Facility of Instrument, Institute of Basic Medical Sciences, Chinese Academy of Medical Sciences/School of Basic Medicine, Peking Union Medical College, Beijing, China; The Ohio State University, UNITED STATES

## Abstract

High-fat diet (HFD) leads to the development of obesity accompanied by insulin resistance, which increases the risk of type 2 diabetes mellitus and cardiovascular disease. Brown adipose tissue (BAT) plays an essential role in energy metabolism, thus it will give us promising treatment targets through elucidating underlying mechanisms of BAT in obesity. In this study, female C57BL/6J mice were fed HFD or normal diet (ND) for 22 weeks. Hyperinsulinemic-euglycemic clamp was performed to evaluate insulin sensitivity, which was independently correlated with obesity. Using isobaric tag for relative and absolute quantification (iTRAQ) coupled with 2D LC-MS/MS, we quantitated 3048 proteins in BAT. As compared HFD with ND, we obtained 727 differentially expressed proteins. Functional analysis found that those proteins were mainly assigned to the pathway of mitochondrial function. In this pathway, carnitine O-palmitoyltransferase 2 (CPT2), uncoupling protein 1 (UCP1) and apoptosis-inducing factor 1 (AIF1) were up-regulated significantly by HFD, and they were confirmed by western blotting. The results indicated that HFD might induce the apoptosis of brown adipocytes via the up-regulated AIF1. Meanwhile, HFD also stimulated fatty acid β-oxidation and raised compensatory energy consuming through the increases of CPT2 and UCP1, respectively. However, the apoptosis of brown adipocytes might weaken the compensatory energy expenditure, and finally contribute to overweight/obesity. So, preventing the apoptosis of brown adipocytes may be the key target to treat obesity.

## Introduction

Obesity is becoming increasingly prevalent in the world and it causes a variety of health problems, such as insulin resistance, dyslipidemia, hypertension and chronic inflammation, all of which contribute to high rates of mortality and morbidity [1,2]. The basic characteristics of obesity are considered as excessive adipose tissue accumulation. There are two main types of adipose tissue in mammals: white adipose tissue (WAT) and brown adipose tissue (BAT), both of which have different physiological roles and can be distinguished by their morphology and metabolic features. WAT mainly acts as a fat reservoir and an endocrine organ in energy homeostasis. As compared with WAT, BAT plays a prominent part in energy expenditure and heat production. BAT is rich in blood vessels and made up of cells that contain numerous mitochondria [3]. The mitochondria, marking the major features of BAT, exert uncoupling of oxidative phosphorylation from ATP synthesis, then convert energy into heat. This process is mainly mediated by UCP1, expressed in the inner membrane of mitochondria of brown adipocytes [4]. It was ever thought that BAT mainly existed in small mammals and human neonates for non-shivering thermogenesis. However, several studies have observed that functional BAT existed in healthy human adults and its activity was decreased when men became overweight or obese [5,6,7,8,9]. Thus, further study of the pathophysiology of BAT might discover promising targets for the treatment of obesity.

For comprehensive understanding the role of adipose tissue in obesity, a few attempts have been made to reveal WAT or BAT proteomics and have identified some essential regulators involved in obesity. Schmid and coworkers have taken a proteomic study by two-dimensional gel electrophoresis (2-DE) coupled with tandem mass spectrometry in BAT of C57BL/6 mice. They identified 12 significantly different proteins between obese and lean mice, while several proteins belonged to the pathway of stress and redox [10]. Using 2-DE and MALDI-TOF-MS, Okita and colleagues performed a study to compare the expression of proteins between caloric restriction group and control group in WAT and BAT of rats. They found 7 and 9 differentially regulated proteins in WAT and BAT respectively. In addition, ATP-citrate synthase (ACLY), as a primary enzyme involved in lipogenesis, was up-regulated by caloric restriction. It demonstrated that caloric restriction might change the activity of enzymes associated with energy metabolism [11]. With the same approach, Joo and colleagues identified 60 and 70 differentially expressed proteins in BAT and WAT respectively. They revealed that the expression of several thermogenic enzymes and lipogenic enzymes were varied in adipose tissues of obese prone and obese resistant rats [12].

Despite the success of studies mentioned above, molecular mechanisms, particularly the role of BAT, related to obesity remain to be elucidated. In recent years, with the development of proteomic technology, high-resolution and high-throughput mass spectrometry was used to identify and quantify proteome of different tissues or cells [13,14,15]. Therefore, to understand the function of BAT in obesity, we performed comparative proteome analysis of BAT between obese and normal female mice using iTRAQ-coupled 2D LC-MS/MS.

## Materials and Methods

### Animals

All animals were handled according to the Standards for Laboratory Animals (GB14925-2001) and the Guideline on the Humane Treatment of Laboratory Animals (MOST 2006a) established by the People’s Republic of China. The two guidelines were conducted in adherence to the regulations of Institutional Animal Care and Use Committee (IACUC) and all animal procedures were approved by IACUC (approval number: SCXKBeijing- 2009-0004). All efforts were made to minimize suffering. Six- to eight-week-old female C57BL/6J mice were purchased from HFK Bioscience Laboratories (Beijing, China). All mice were maintained under SPF conditions and 12h light/dark cycle at 23±2°C, with free access to water and diet. Mice were divided into two groups: one was fed standard chow (SC; 10% lipids) diet and another group was fed high-fat (HF; 45% lipids) diet for 22 weeks. Food and water intake for 24 hours and body weight (once per week) were dynamically monitored.

### Oral glucose tolerance test (OGTT) and hyperinsulinemic-euglycemic clamp

For OGTT, 5 h fasted mice were given glucose (2.0 g/kg body weight, I.G.). Blood from the tail (10 μL) was taken every 30 min (0 min to 120 min after glucose was given). Blood glucose concentrations were measured by glucose oxidase method [10]. Plasma levels of total cholesterol (TC) and triglyceride (TG) were tested by enzymic method (Biosino bio-technology & science Inc, China). Hyperinsulinemic-euglycemic clamp studies were performed at 22 weeks. With overnight fasted, mice were anesthetized with 80 mg/kg pentobarbital sodium, and inserted a single implantation tubing into the right jugular vein for infusion of insulin or glucose. Mice were placed on their back on a warming surface (37°C) platform during clamp. After the adaptation period, hyperinsulinemic-euglycemic clamp was conducted and lasted for 120 min. Indwelling catheter infused with constant rate of human insulin (20 mIU/kg/min) and variable rate of glucose solution (25%, w/v) to maintain blood glucose level at 5.5±0.5 mmol/L. The tail tips were cut off for testing blood glucose every 5 min. The insulin sensitivity was measured by glucose infusion rate (GIR) during the last 80 min [16]. At the end of the study, all of the mice were decapitated.

### Preparation of BAT samples

Interscapular BAT of mice in ND group (n = 6) and HFD group (n = 6) were excised and washed with a cold saline solution. Two groups of BAT were snap frozen in liquid nitrogen and kept at -80°C until analysis. Tissues were cut into small pieces and lysed in buffer solution containing 7 M urea, 2 M thiourea, 65 mM DTE, 83 mM Tris (Sigma-Aldrich, St. Louis, MO, USA) and then homogenized with a homogenizer (IKA R104, Janke& Kunkel KG.IKA-werk, Germany) on ice. Extracts were centrifuged at 20 000 g for 10 min at 4°C, and the supernatant was then stored at -80°C. Protein content of adipose tissues was determined by the Bradford method with Bradford reagents (Thermo Fischer Scientific, USA).

Total BAT proteins of six mice fed ND were pooled at the same amount, and BAT proteins of six mice fed HFD were also pooled with the same method. Samples were processed through columns (micro Bio-spin, nanosep 10k omega; PALL, USA) according to the manufacturer’s instructions. Each of the 3 samples (100 μg in total) in one group was deoxidized with 20 mM DTT, alkylated with 50 mM IAA and digested with trypsin. After digestion, peptides were desalted with HLB 3cc extraction cartridges (Oasis, Waters, Ireland), cleaned up with 500 μL 0.1% formic acid and eluded with 500 μl 100% ACN. Peptide elution was vacuum-dried and stored at -80°C.

### iTRAQ labeling

BAT peptide samples were respectively labeled with iTRAQ reagent as follows: The digested ND samples were considered as internal standard. The internal standard and HFD samples were labeled by 114, 115 iTRAQ. Labeling was performed according to the manufacturer’s protocol (ABsciex, Massachusetts, USA) [17]. The ND and HFD samples were mixed into one sample at the same amount and lyophilized.

### LC-MS/MS

The pooled mixture from labeled samples was first fractioned by high-pH RPLC column from Waters (4.6 mm×250 mm, C18, 3 μm). The samples were loaded onto the column in buffer A2 (pH = 10). The eluted gradient was 5–90% buffer B2 (90%ACN; pH = 10, flow rate, 1 mL/min) for 60 min. The eluted peptides were collected as a fraction per minute, and the 60 fractions were pooled into 20 samples. Each sample was analyzed by RP C18 self-packing capillary LC column (75 μm×100 mm, 3 μm). The eluted gradient was 5–30% buffer B1 (0.1% formic acid, 99.9% ACN; flow rate, 0.5 μL/ min) for 100 min. Triple TOF 5600 were used to analyze the sample. The MS data were acquired with high sensitivity mode using the following parameters: 30 data-dependent MS/MS scans per every full scan; full scans was acquired at resolution 40,000 and MS/MS scans at 20,000; 35% normalized collision energy, charge state screening (including precursors with +2 to +4 charge state) and dynamic exclusion (exclusion duration 15 s); MS/MS scan range was 100–1800 m/z and scan time was 100 ms.

### Database search

The MS/MS spectra were respectively searched against the SwissProt mouse database from Uniprot website (http://www.uniprot.org) using Mascot software version 2.3.02 (Matrix Science, UK). Trypsin was chosen as cleavage specificity with a maximum number of allowed missed cleavages of two. Carbamidomethylation (C) and iTRAQ 4-plex label was set as a fixed modification. The searches were performed using a peptide and production tolerance of 0.05 Da. Scaffold was used to further filter the database search results by decoy database method. The following filter was used in this study, 1% false positive rate at protein level and each protein with 2 unique peptides. After filtering the results by above filter, the peptide abundances in different reporter ion channels of MS/MS scan were normalized. The protein abundance ratio was based on unique peptide results. Proteins of adipose tissue with a fold change <1.5 were excluded, in order to reduce technical variability.

### Gene Ontology (GO) functional analysis

All differential proteins identified by two approaches were assigned their gene symbol via the PANTHER database (Protein Analysis through Evolutionary Relationships, http://www.pantherdb.org/). Protein classification was performed based on their functional annotations using GO for cellular component, biological process, and molecular function. When more than one assignment was available, all of the functional annotations were considered in the results.

### IPA network analysis

All differential proteins were used for pathway analysis. For this purpose, the SwissProt accession numbers were inserted into the Ingenuity Pathway Analysis (IPA) software (Ingenuity Systems, Mountain View, CA). This software categorizes gene products based on the location of the protein within cellular components and suggests possible biochemical, biological and molecular functions. Furthermore, proteins were mapped to genetic networks available in the Ingenuity and other databases and ranked by score. These genetic networks describe functional relationships between gene products based on known interactions in literature. Through the IPA software, the newly formed networks were associated with known biological pathways.

### Western blotting

BAT protein was lysed in buffer solution containing 7 M urea, 2 M thiourea, 65 mM DTE, 83 mM Tris (Sigma-Aldrich, St. Louis, MO, USA). Protein content of adipose tissues was determined by the Bradford method with Bradford reagents (Thermo Fischer Scientific, USA). Protein solution and loading buffer were mixed in proportion followed the instructions and boiled for 7 min. Equal amounts of proteins (25 μg) were then subjected to NuPAGE 4–12% Bis-Tris gel for electrophoresis and transferred to Polyvinylidene Fluoride Membranes (0.45 μm). Membranes were blocked with 5% nonfat dry milk in TBS (10 mM Tris-HCl, 150 mM NaCl, pH 7.6) for 1.5 h at room temperature. The membranes were probed with primary antibody overnight at 4°C. The antibodies were used as follows: anti-rabbit CPT2 (1:1000), AIF1 (1:500), UCP1 (1:300) (all Abcam, Cambridge, U.K.), anti-mouse β-actin (1:4000; Sigma–Aldrich, U.S.A). After washing with TBST, the membranes were incubated with HRP conjugated goat anti-rabbit IgG (1:5000; Earthox LLC., San Francisco, California, U.S.A) and anti-mouse IgG secondary antibody (1:3000; Cell Signaling Technology, Inc., Danvers, U.S.A), for 1.5 h at room temperature. After washing with TBST, the membranes were incubated with chemiluminescent HRP Substrate (Millipore Corporation, Billerica, U.S.A). Western blot was analyzed by scanning with LAS4000 (Fujifilm, Tokyo, Japan) and the data were analyzed using Image J software. β-actin was used as a normalization control. Duplicate experiments were carried out for all proteins.

### Data analysis and statistics

Data derived from six mice in ND and HFD group were presented as mean ± SEM and were examined using Student’s t-test. Differences with p values<0.05 were considered to be statistically significant.

## Results

**Fig 1:**
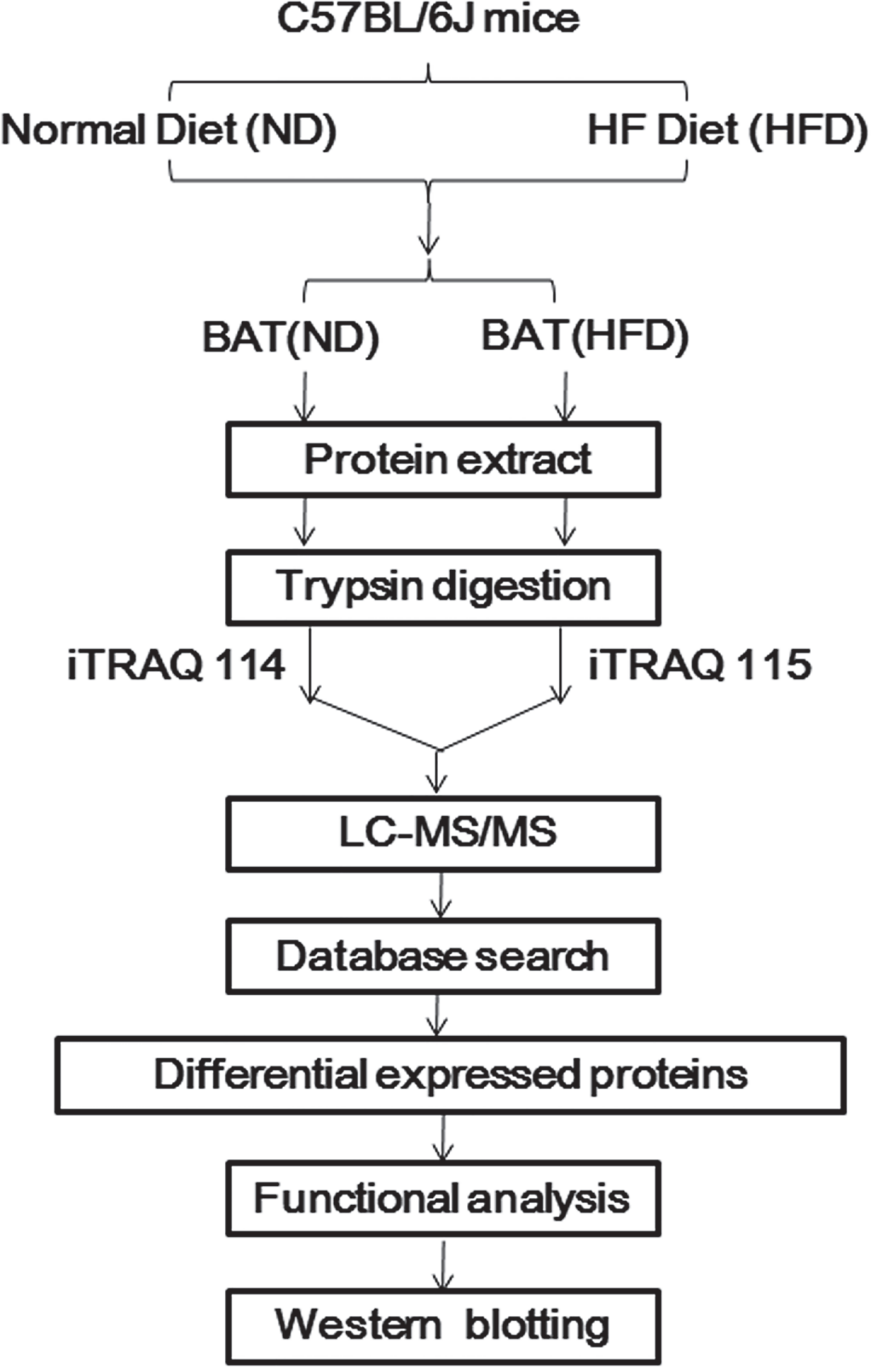
Work flow of establishing obese mice model and proteomic analysis of BAT proteins. Female C57BL/6J mice were fed normal diet (ND) or high-fat diet (HFD) start at 6–8 weeks of age. Glucose tolerance test (OGTT) and hyperinsulinemic-euglycemic clamp were applied to assess the insulin sensitivity in mice after 22 weeks. The levels of blood lipid were tested as the auxiliary evaluation indexes of metabolic state. Proteins of BAT in ND and HFD group were quantified via iTRAQ-coupled 2D LC-MS/MS. After comparing the differential expressed proteins between HFD group and ND group, functional analysis of those differential proteins of BAT indicated the important role of BAT in obesity. Finally, western blot analysis was performed to validate the variation of three key differential proteins.

In our study, female C57BL/6J mice were fed normal diet (ND) or high-fat diet (HFD) start at 6–8 weeks of age. At 22 weeks, we applied OGTT and hyperinsulinemic-euglycemic clamp to assess glucose regulation states and insulin sensitivity in mice, respectively. Moreover, the levels of blood lipid were tested as evaluation indexes of metabolic state. In order to obtain the global proteome of BAT in obesity, we identified and quantified the expression of total proteins of BAT via iTRAQ-coupled 2D LC-MS/MS. Then we compared the differential expressed proteins between HFD group and ND group in BAT and furthermore, analyzed their functions. Finally, western blot analysis was performed to validate the variation of three key differential proteins (Fig. 1).

### Weight gain, dyslipidemia, glucose intolerance and impaired insulin sensitivity induced by HFD

**Fig 2:**
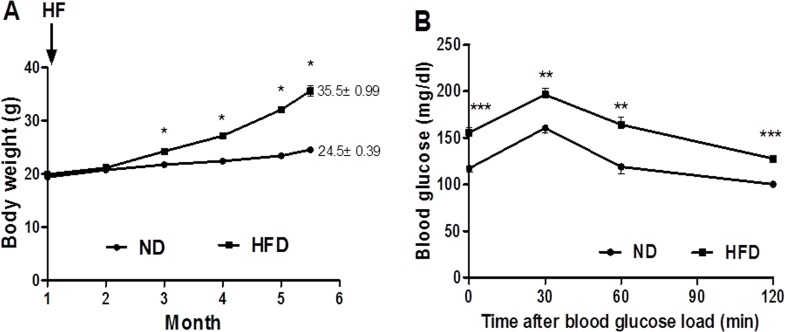
Body weight and metabolic status of mice were affected by HFD. HFD significantly altered the body weight (A), and induced glucose intolerance (B) in C57BL/6J mice. Black circle = Normal diet group, black squares = High-fat diet group. Data are mean ± SEM (n = 6), *p<0.05, **p<0.01, ***p<0.001.

C57BL/6J mice were fed HFD and ND respectively. At 22 weeks, body weight of mice fed HFD was significantly increased (35.5±0.99 g) as compared to the ND group (24.5 ± 0.39 g) (Table 1; Fig. 2A), and gonadal WAT weight of HFD group per body weight was significantly higher than that of ND group (Table 1). However, the interscapular BAT weight of HFD group per body weight was significantly lower than that of ND group (Table 1). The plasma levels of TC and TG were strongly higher in HFD group (Table 1).

**Table 1:** Body weight, adipose tissue weight and metabolic profile of female C57BL/6J mice fed ND or HFD for 22 weeks (mean ± SEM).

	ND	HFD	p-value
Body weight (g)	24.5±0.39	35.5±0.99	<0.001
WAT weight/ body weight (%)	2.15±0.41	4.92±0.40	<0.001
BAT weight/ body weight (%)	0.26±0.04	0.13±0.02	0.012
Cholesterol (mg/dl)	38.11±0.99	83.89±2.29	<0.001
Triglyceride (mg/dl)	62.10±1.52	79.48±2.67	0.003
Glucose infusion rate (mg·kg^−1^·min^−1^)	52.332±5.697	14.748±1.478	0.0014

For the preliminary assessment of glucose tolerance, OGTT was performed on the two groups of mice at 22 weeks, and the area under curve of blood glucose of HFD group (324.15±3.97 mg/dl·min) is much greater than that of ND group (249.04±7.53 mg/dl·min) (p<0.001) (Fig. 2B).

Three days after OGTT, insulin sensitivity was measured by hyperinsulinemic-euglycemic clamp, which is the “gold standard” method for assessing insulin sensitivity in vivo. The mean of GIR during the last 80 min revealed that HFD decreased insulin sensitivity in mice significantly as compared to ND group (Table 1). Thus, C57BL/6J mice were successfully led to obesity with impaired insulin sensitivity by HFD for 22 weeks.

### Protein expression profiling of BAT in mice fed HFD or ND

Using LC-MS/MS, we identified 3486 proteins in BAT as the following criteria were required in scaffold: two or more high confidence unique peptides had to be identified; false positive rate of the identification of protein or peptide was less than 1%. Among 3486 proteins in BAT, we quantified 3212 proteins across replicate experiments (Table 2). In order to evaluate relative variability of two run assays with LC-MS/MS, we calculated the coefficient of variation (CV) for two runs. 88.5% of proteins, whose CV were ≤20%. To diminish technical error, proteins with CV >20% were excluded (S1, S2 Figs.). Finally, we obtained 2696 proteins in BAT. As compared HFD to ND, we obtained 727 differentially expressed proteins in BAT, since the fold change ≥1.5 (fold change is the ratio of intensity of protein expression in HFD to ND adipose tissue) was considered as a threshold to minimize biological and technical errors (S3 Fig.).

**Table 2:** Amount of proteins, peptides and spectrums identified and quantified by LC-MS/MS in BAT from two run assays.

	Qualitative			Quantitative		
	Run1	Run2	Total	Run1	Run2	Total
Proteins	3444	3458	3486	3125	3117	3212
Peptides	21737	21630	25105	20579	20476	23821
Spectrums	87190	86613	173803	85439	84062	169501

### Comprehensive functional assessment of the quantified proteins

**Fig 3:**
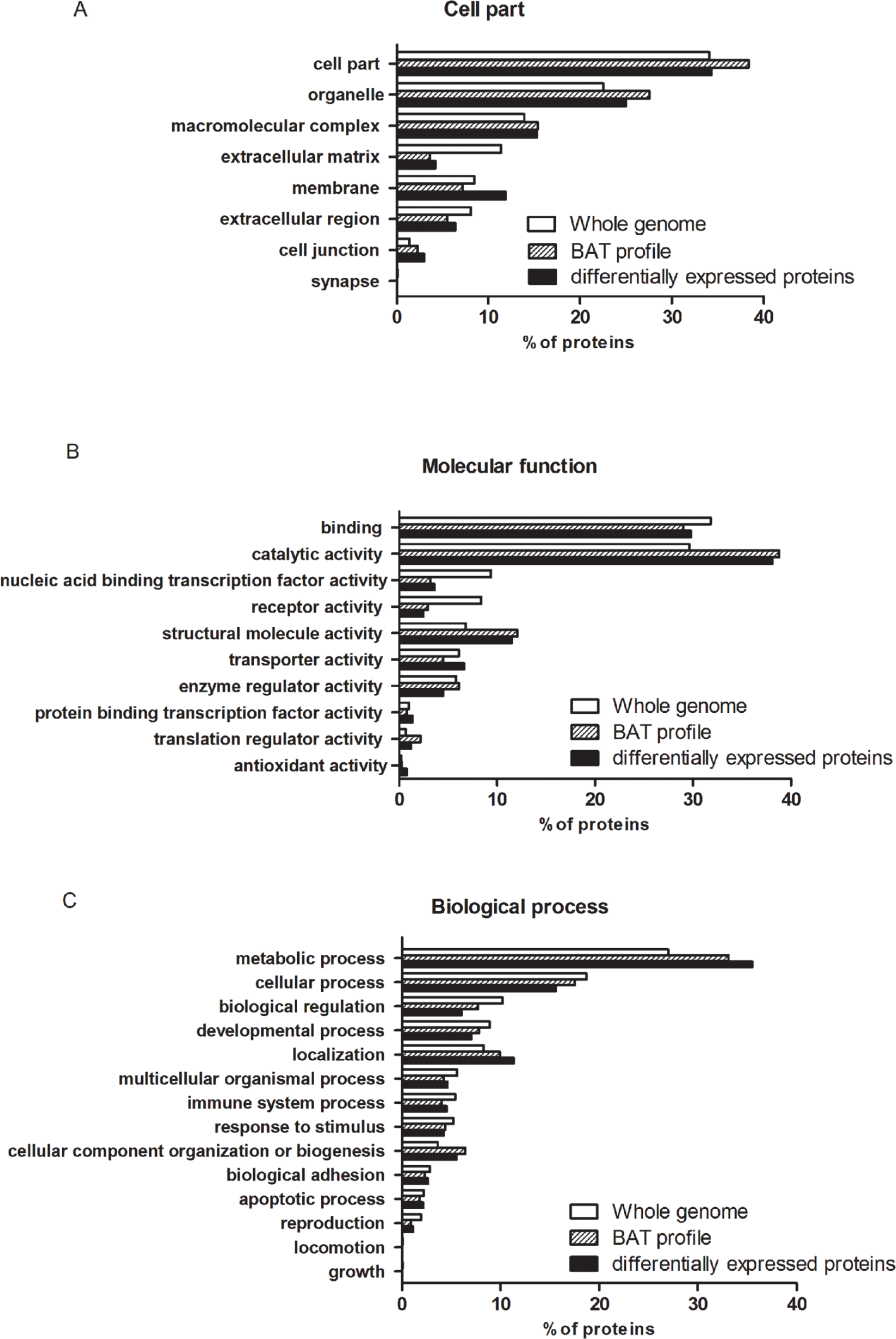
Differential proteins between HFD and ND group in BAT were analyzed through the PANTHER classification system. (A) Cell Component of whole genome, BAT protein profile and the differentially expressed proteins in BAT. (B) Molecular Function assigned to whole genome, BAT protein profile and the differentially expressed proteins in BAT. (C) Biological processes assigned to whole genome, BAT protein profile and the differentially expressed proteins in BAT.

These differential proteins were preliminary analyzed according to GO database (http://www.pantherdb.org/). 34.3% of differential proteins were assigned to cell part. We also observed that HFD increased the expression of membrane proteins (Fig. 3A). The molecular function and biological process of the differentially expressed proteins were also annotated. 38.1% of differential proteins were assigned to catalytic activity in BAT and 11.5% of differential proteins involved in structural molecule activity were regulated (Fig. 3B). It indicated that BAT was rich in proteins which involved in catalytic activity and structural molecule activity, as compared BAT profile with whole genome. HFD increased the amount of proteins which were assigned to transporter activity. Moreover, the proportion of differentially expressed proteins (35.5%) involved in metabolic process was higher than which of total BAT proteins (33.1%) and whole genome profile (27.0%) (Fig. 3C). It suggested that HFD stimulated the activity of proteins that related to metabolism.

In addition, based on GO database, proteins which involved in metabolic process were classified into different groups. We found that proteins assigned to fatty acid metabolism were regulated significantly by HFD. Those proteins including long-chain-fatty-acid—CoA ligase 1 (ACSL1), long-chain fatty acid transport protein 1 (Slc27A1), Very long-chain acyl-CoA synthetase (Slc27A2), trifunctional enzyme subunit beta, mitochondrial (HADHB), Carnitine O-palmitoyltransferase 1b, mitochondrial (CPT1b) and isoforms Carnitine O-palmitoyltransferase 2, mitochondrial (CPT2) were up-regulated. On the other hand, proteins involved in fatty acid biosynthesis—ATP-citrate synthase (ACLY), acetyl-CoA carboxylase 1 (ACACA) and fatty acid synthase (FASN)—were down-regulated by HFD.

Enzymes for glucose metabolism in BAT were also altered significantly by HFD. The key enzymes, located in cytoplasm, involved in glycolysis, such as hexokinase-1 (HXK1) and 6-phosphofructokinase, muscle type (K6PF), were up-regulated. However, the rate-limiting enzymes related to tricarboxylic acid cycle (TCA cycle) in mitochondria, such as pyruvate dehydrogenase protein X component, mitochondrial (PDHX) and pyruvate dehydrogenase E1 component subunit beta, mitochondrial (PDHB) were down-regulated in BAT.

**Fig 4:**
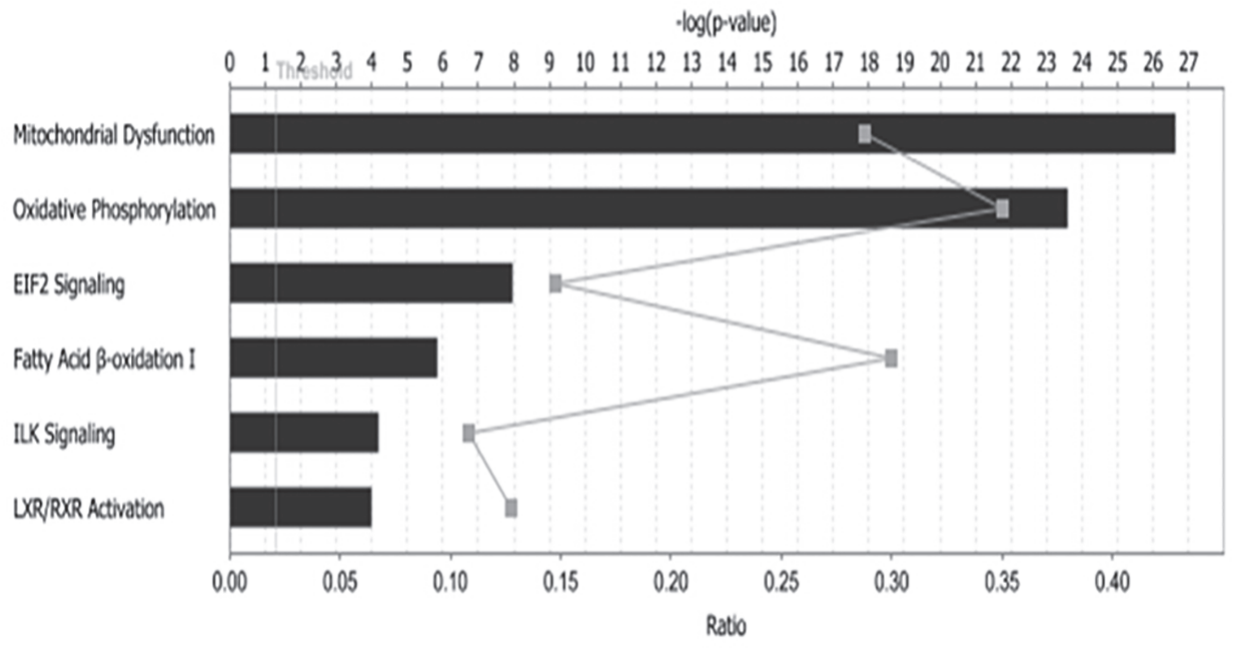
Ingenuity Pathway Analysis was performed to analyze the function of proteins altered by HFD. Differentially expressed proteins in BAT were characterized, and the top 5 of statistically significant pathways were exhibited.

Furthermore, we applied Ingenuity Pathway Analysis (http://www.ingenuity.com) to analyze the function of proteins altered by HFD. Differentially expressed proteins in BAT were characterized, and the top five of statistically significant pathways were associated with metabolism (Fig. 4). In the pathway of mitochondrial dysfunction (-log (p value) was 28): uncoupling protein 1 (UCP1), involved in energy metabolism, was up-regulated in HFD group; apoptosis-inducing factor 1 (AIF1), voltage-dependent anion-selective channel protein (VDAC) and BCL2/adenovirus E1B 19 kDa protein-interacting protein 3 (BNIP3) were also up-regulated and related to mitochondria-mediated apoptosis[18,19]; whereas Superoxide dismutase [Cu-Zn] (SOD1) and Glutathione peroxidase 1 (GPX1), involved in oxidative stress[20,21], were down-regulated in HFD group.

### Validation of proteomic data by western blotting

**Fig 5:**
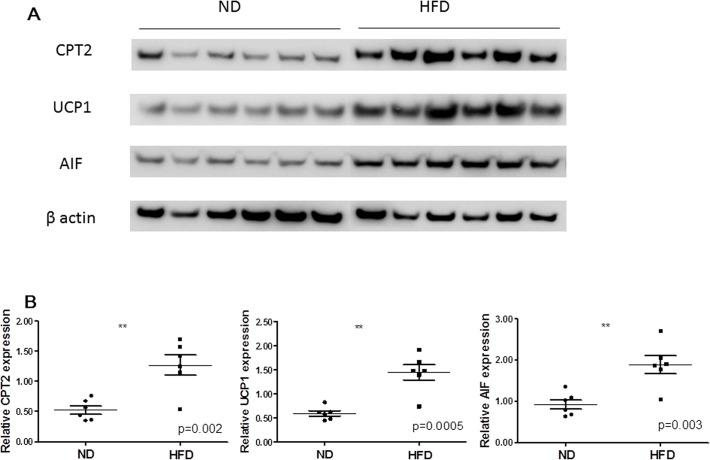
Western blot analysis compared expression of CPT2, UCP1 and AIF1 in BAT between HFD and ND group. (A) Gel images of western blotting between HFD and ND group of six mice. (B) β-actin was used as a normalization control. Compared normalized density values from blots between HFD and ND group. **p<0.01.

On the basis of comprehensive functional analysis of differential expressed proteins, which involved in energy metabolism and function of mitochondria were significantly regulated. To further confirm our observations, western blotting analysis was performed for the three key potential proteins, including CPT2, UCP1 and AIF1 (Fig. 5A, B). All of those proteins were up-regulated by HFD, and the differences between two groups were statistically significant. As shown in Table 3, the expression levels of three proteins were consistent with those of the LC-MS/MS study.

**Table 3:** Fold change of BAT proteins between HFD and ND group (mean ± SEM) measured by iTRAQ coupled with LC-MS/MS and western blotting (normalized to β-actin).

Protein	iTRAQ (HFD/ND)	Western blotting (HFD/ND)	
	Fold change	Fold change	p value
CPT2	2.15±0.071	2.67±0.458	0.002
UCP1	3.75±0.071	2.57±0.432	0.0005
AIF1	1.65±0.071	2.25±0.439	0.003

CPT2, Carnitine O-palmitoyltransferase 2; UCP1, uncoupling protein 1

AIF1, apoptosis-inducing factor 1.

## Discussion

Obesity is the imbalance of income and expenditure of energy. Adipose tissue plays a central role in the balance of energy metabolism. WAT mainly acts as a fat storage, prone to obesity, while BAT is a major organ that releases the energy through the heat production, against obesity. However, mechanisms of the development of obesity via dysfunction of BAT remain to be further researched. In our study, we developed the obese C57BL/6J mice model with metabolic disorder by HFD. For a large-scale exploration of variation in BAT proteome under the state of obesity, we performed a comparative analysis of the proteins expression profiles of BAT in mice fed HFD and ND by iTRAQ and 2D LC-MS/MS. High-resolution of LC-MS/MS provided us a higher-performance proteome map. Those differential expressed proteins indicated that disturbance of fatty acid metabolism and mitochondrial dysfunction might be involved in the pathophysiology of obesity.

### HFD influenced fatty acid and glucose metabolism in BAT

**Fig 6:**
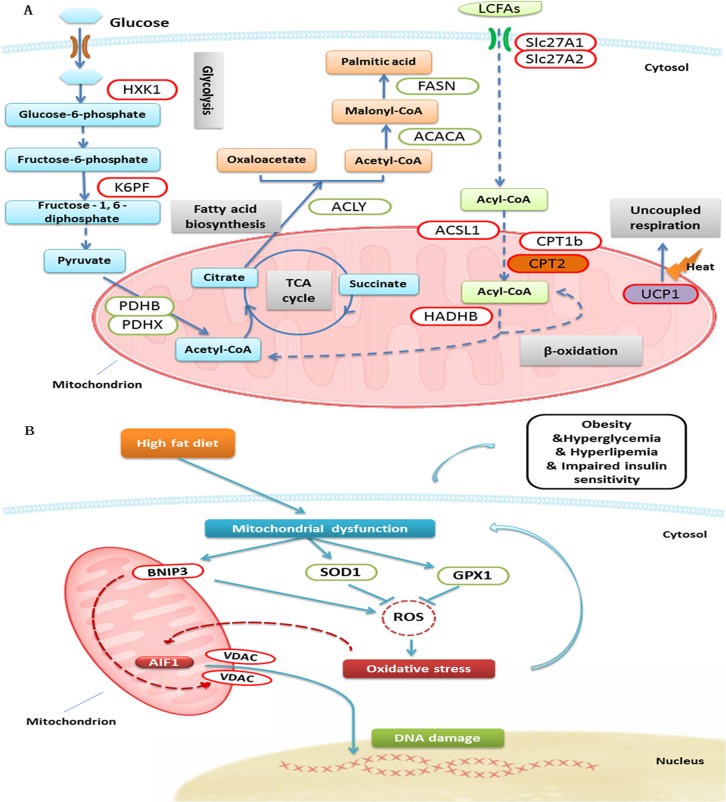
Differential proteins in BAT were involved in several essential biological processes. (A) As compared HFD with ND group in BAT of mice, some differential proteins were key enzymes involved in glycolysis, tricarboxylic acid cycle, fatty acid biosynthesis and β-oxidation, and uncoupled respiration. Red and green circles with white fill represent up- and down-regulated proteins, respectively. CPT2, UCP1 were validated with western blot analysis. (ACACA, acetyl-CoA carboxylase 1; ACLY, ATP-citrate synthase; ACSL1, long-chain-fatty-acid—CoA ligase 1; CPT1b, Carnitine O-palmitoyltransferase 1b, mitochondrial; CPT2, isoforms Carnitine O-palmitoyltransferase 2, mitochondrial; FASN, fatty acid synthase; HADHB, trifunctional enzyme subunit beta, mitochondrial; HXK1, hexokinase-1; K6PF, 6-phosphofructokinase, muscle type; PDHB, pyruvate dehydrogenase E1 component subunit beta, mitochondrial; PDHX, pyruvate dehydrogenase protein X component, mitochondrial; Slc27A1, long-chain fatty acid transport protein 1; Slc27A2, very long-chain acyl-CoA synthetase; UCP1, uncoupling protein 1). (B) Compared HFD with ND group in BAT of mice, differential proteins which involved in oxidative stress and cell apoptosis were exhibited as a network. Disturbance of metabolism induces mitochondrial dysfunction, which will cause oxidative stress and cell apoptosis. Oxidative stress in turn will lead to mitochondrial dysfunction and then form a circle. AIF1 was validated with western blot analysis. (AIF1, apoptosis-inducing factor 1; BNIP3, BCL2/adenovirus E1B 19 kDa protein-interacting protein 3; GPX1, Glutathione peroxidase 1; ROS, reactive oxygen species; SOD1, Superoxide dismutase [Cu-Zn]; VDAC, voltage-dependent anion-selective channel protein).

In current study, mice fed HFD were developed to hyperglycemia and hyperlipidemia. With LC-MS/MS, fatty acid metabolism in BAT, we found that HFD inhibited the fatty acid biosynthesis (ACACA, ACLY, FASN) in BAT. This was in agreement with the previous study [11]. Moreover, we found that the key enzymes for fatty acid β-oxidation, such as CPT1b and CPT2 were up-regulated in BAT. CPT1, located in the outer mitochondrial membrane, is the key enzyme for transport of long-chain fatty acids (LCFAs) into mitochondria [22]. It has been reported that CPT1a and CPT1b were important elements for regulation of energy homeostasis in heart and skeletal muscle [23]. CPT2, expressed in the inner mitochondrial membranes, functions as a transporter of LCFAs and convert it to acyl-CoAs. Evidence has shown that CPT2 deficiency caused myolysis attacks for the fact that skeletal muscle was provided energy mainly by LCFAs [22]. Thus, the up-regulated CPT1b and CPT2 indicated that HFD might increase the energy expenditure in BAT (Fig. 6A).

For glucose metabolism, the regulation of associated key-enzymes was varied in BAT by HFD. The enzymes for glycolysis (HXK1, K6PF) located in cytoplasm were up-regulated, while the key enzymes associated with TCA cycle (PDHB, PDHX) in mitochondria were down-regulated [12]. It suggested that HFD might impair the glucose metabolism in mitochondria of BAT (Fig. 6A).

The study performed by Ho and colleagues [17], presented the correlation between fatty acid metabolism and glucose intolerance in prediabetic mice. They researched the different expressions of WAT membrane proteins between HFD group and control group in 2, 6 and 8 month with iTRAQ approach. The results showed that CPT1b was up-regulated in WAT by HFD, while CPT1a and CPT2 were unaffected. Similar to the study, we found that CPT1b was up-regulated in BAT, but we also identified the up-regulation of CPT2. As compared with Ho’s study, it implied that, in BAT, CPT2 might have different reaction to HFD.

### HFD affected energy metabolism in BAT

Mitochondria are the main organelles that produce energy in cells [24]. Energy metabolism is prominent in brown adipocytes, which are rich in mitochondria. UCP1 is mainly expressed in the mitochondria of BAT, and uncouples from ATP production in mitochondrial respiratory chain to dissipate energy. In adult human, BAT can be altered by variable factors, such as gender, year of age, body mass index, circumstance temperature and medicine [7,9,25,26]. Recent evidences have showed that excess caloric intake increased the expression of UCP1 in BAT. As the extension of time with high-calorie diet, the relative level of UCP1 could reach a peak, and later it gradually declined [27]. Here, using iTRAQ coupled with 2D LC-MS/MS, we observed that UCP1 in BAT was up-regulated by HFD for 22 weeks in mice. Furthermore, we confirmed it with western blot analysis (Fig. 5A, B).

In Ho’s study, they also observed the up-regulation of UCP1 in WAT. It is interesting that the fold change of UCP1 in 6 month was higher than that of 2 and 8 month. It indicated that, in our study, UCP1 in BAT reacted with a compensatory rise in HFD group at 22 weeks (Fig. 6A). Those protective proteins and relative actions mentioned above didn’t resist the development of obesity, thus it implied that there might be some other factors contributing to the development of obesity.

### HFD increased apoptosis of brown adipocytes

HFD can induce obesity, which associated with hyperglycemia, hyperlipemia and impaired insulin sensitivity. Previous studies showed that long-term metabolic disorder affected the function of mitochondria, and decreased the activity of enzymatic antioxidant, such as SOD1 and GPX1 [28]. In addition, there were evidences revealed that increased BNIP3 was associated with the generation of reactive oxygen species (ROS) [29]. Thus, ROS would be overload produced and thereby causing oxidative stress, which led to apoptosis [30,31]. Mitochondria play a key role in the process of oxidative stress and apoptosis pathways [32]. In those pathways, we identified the up-regulation of AIF1, VDAC and BNIP3 by accompanied with the down-regulation of SOD1 and GPX1 via LC-MS/MS (Fig. 6B).

AIF1 has oxidoreductase activity and acts as a caspase-independent mitochondrial effector of apoptotic cell death [18,33]. Many laboratories have demonstrated that AIF, as a death effector, can transfer from mitochondrion to the nucleus to execute apoptosis with chromatin condensation and oligonucleosomal DNA fragmentation; while caspases can be inactive in this situation [18,34,35,36,37,38]. VDAC, as a mitochondrial permeability transition pore (mPTP) component, is a protein forming the aqueous pore channel in the mitochondrial outer membrane [39]. VDAC1 plays a critical role in regulating mitochondrial Ca^2+^ homeostasis and energy metabolism [40]. In addition, VDAC2 and VDAC3 as well as VDAC1 also acts as a key point in apoptosis, involved in regulation of mitochondrial membrane permeability to balance the apoptosis and the release of AIF [41,42,43,44,45]. Besides, BNIP3 can regulate the opening of mPTP to mediate mitochondrial dysfunction and apoptosis [46,47,48].

In the present study, the down-regulation of SOD1 and GPX1 indicated that brown adipocytes in HFD group were suffered from oxidative stress. The increased AIF1, VDAC and BNIP3 revealed that high-fat diet might lead to the apoptosis of brown adipocytes. As expected, western blot analysis proved this hypothesis that AIF1 was unregulated by 2.25 times in HFD group (Fig. 5A, B).

As compared with Ho’s study [17], they also identified and quantified AIF1 in membrane proteins of WAT. However, it was not up-regulated significantly. Combined with our results, those might explain that HFD induce the apoptosis of BAT, but not WAT. Thus, it is likely to explain that HFD led to the excessive accumulation of WAT, while decrease BAT mass, thereby inducing obesity.

Those discoveries were limited to the female, yet these may provide clues to the male. Cypess and coworkers identified BAT in adult human; in addition, they found that the prevalence of detectable BAT was higher in women, while the median mass and activity of BAT were similar in both genders [9]. Yang et al. reported that female and male mice had different response to HFD. Food intake and body composition were different between both sexes [49]. Thus, for reducing the influence of confounding factors, we selected only female mice in our study. Further study is required to elucidate whether there are differences between male and female mice in terms of the pathophysiology of BAT.

## Conclusions

**Fig 7:**
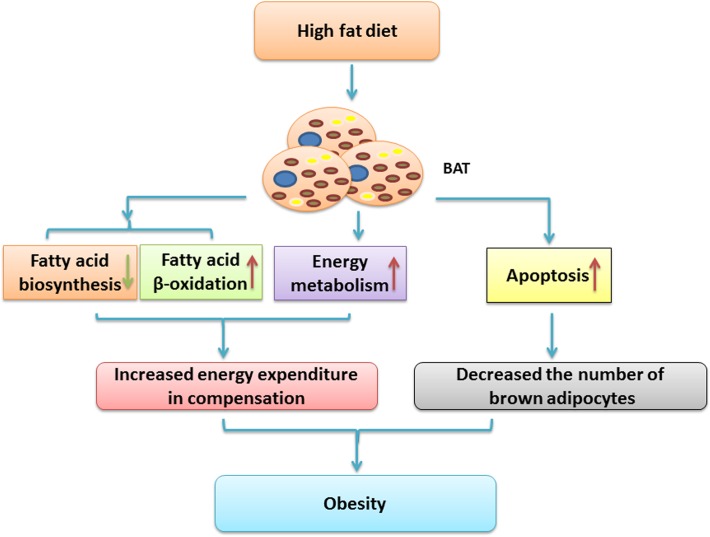
Proposed schematic diagram of BAT in obesity and impaired insulin sensitivity induced by HFD. It is likely that HFD activates the catabolism of fatty acid and the compensatory energy expenditure for anti-obesity through the up regulation of CPT2 and UCP1 in BAT, respectively. In addition, HFD also induces the apoptosis of brown adipocytes via the up regulation of AIF1, to weaken its anti-obesity effect. Attenuating compensatory action of BAT might cause imbalance of energy metabolism in BAT and WAT, and contribute to the development of obesity and impaired insulin resistance.

In summary, female C57BL/6J mice were successfully induced to obesity and decreased insulin sensitivity by HFD for 22 weeks. We applied comparative proteomics to analyze the differential expression proteins in BAT of C57BL/6J mice fed HFD and ND. With iTRAQ-coupled 2D LC-MS/MS, we found that HFD not only inhibited glucose metabolism and the synthesis of fatty acid, but also activated the catabolism of fatty acid in BAT through the up-regulation of CPT2. Besides, HFD also activated the compensatory energy-consuming process for anti-obesity through the up-regulation of UCP1 in BAT. Above all, HFD might increase the apoptosis of brown adipocytes via a non-classical apoptotic pathway, including AIF1, to weaken its anti-obesity effect. Thus, attenuating the compensatory action of BAT might led to the proliferation of WAT and the reduction of BAT, thereby contributing to the development of obesity and impaired insulin sensitivity (Fig. 7). Further research on AIF1 and upstream mechanisms would illuminate the way of preservation, regeneration and activation of BAT. Moreover, study with multi-period and different degrees of obesity would be more helpful to reveal the targets which can resist the development of obesity and related complications. Thus, preventing the apoptosis of brown adipocytes might be an important clue to treat obesity.

## Supporting Information

S1 Fig The repeatability of experiments with LC-MS/MS was calculated. To evaluate the repeatability of experiments with LC-MS/MS, the correlations of normalized intensity of quantitative proteins between two runs were calculated.
(TIF)

S2 Fig Distribution of the coefficient of variation (CV value) from two run assays in LC-MS/MS was exhibited. In order to minish technical error, proteins whose CV value greater than 0.2 were excluded.
(TIF)

S3 Fig Distribution of ratios between HFD and ND on intensity of protein expression in BAT detected by LC-MS/MS. Fold change greater than 1.5 was considered as a threshold to minimize biological and technical errors.
(TIF)

S1 Dataset Scaffold report for proteins in BAT identified by iTRAQ coupled with 2D LC-MS/MS. (XLSX)

S2 Dataset Scaffold report for peptides in BAT identified by iTRAQ coupled with 2D LC-MS/MS. (XLSX)

## References

[1] Friedman JM (2009) Obesity: Causes and control of excess body fat. Nature 459: 340–342. 10.1038/459340a 19458707

[2] Pospisilik JA, Schramek D, Schnidar H, Cronin SJ, Nehme NT, Zhang X, et al (2010) Drosophila genome-wide obesity screen reveals hedgehog as a determinant of brown versus white adipose cell fate. Cell 140: 148–160. 10.1016/j.cell.2009.12.027 20074523

[3] Enerback S (2010) Human brown adipose tissue. Cell Metab 11: 248–252. 10.1016/j.cmet.2010.03.008 20374955

[4] Krauss S, Zhang CY, Lowell BB (2005) The mitochondrial uncoupling-protein homologues. Nat Rev Mol Cell Biol 6: 248–261. 15738989 10.1038/nrm1592

[5] Klingenspor M (2003) Cold-induced recruitment of brown adipose tissue thermogenesis. Exp Physiol 88: 141–148. 12525862 10.1113/eph8802508

[6] Saito M, Okamatsu-Ogura Y, Matsushita M, Watanabe K, Yoneshiro T, Nio-Kobayashi J, et al (2009) High incidence of metabolically active brown adipose tissue in healthy adult humans: effects of cold exposure and adiposity. Diabetes 58: 1526–1531. 10.2337/db09-0530 19401428 PMC2699872

[7] van Marken LW, Vanhommerig JW, Smulders NM, Drossaerts JM, Kemerink GJ, Bouvy ND, et al (2009) Cold-activated brown adipose tissue in healthy men. N Engl J Med 360: 1500–1508. 10.1056/NEJMoa0808718 19357405

[8] Au-Yong IT, Thorn N, Ganatra R, Perkins AC, Symonds ME (2009) Brown adipose tissue and seasonal variation in humans. Diabetes 58: 2583–2587. 10.2337/db09-0833 19696186 PMC2768171

[9] Cypess AM, Lehman S, Williams G, Tal I, Rodman D, Goldfine AB, et al (2009) Identification and importance of brown adipose tissue in adult humans. N Engl J Med 360: 1509–1517. 10.1056/NEJMoa0810780 19357406 PMC2859951

[10] Schmid GM, Converset V, Walter N, Sennitt MV, Leung KY, Byers H, et al (2004) Effect of high-fat diet on the expression of proteins in muscle, adipose tissues, and liver of C57BL/6 mice. Proteomics 4: 2270–2282. 15274121 10.1002/pmic.200300810

[11] Okita N, Hayashida Y, Kojima Y, Fukushima M, Yuguchi K, Mikami K, et al (2012) Differential responses of white adipose tissue and brown adipose tissue to caloric restriction in rats. Mech Ageing Dev 133: 255–266. 10.1016/j.mad.2012.02.003 22414572

[12] Joo JI, Oh TS, Kim DH, Choi DK, Wang X, Choi JW, et al (2011) Differential expression of adipose tissue proteins between obesity-susceptible and-resistant rats fed a high-fat diet. Proteomics 11: 1429–1448. 10.1002/pmic.201000515 21365757

[13] Forner F, Kumar C, Luber CA, Fromme T, Klingenspor M, Mann M (2009) Proteome differences between brown and white fat mitochondria reveal specialized metabolic functions. Cell Metab 10: 324–335. 10.1016/j.cmet.2009.08.014 19808025

[14] Kim MS, Pinto SM, Getnet D, Nirujogi RS, Manda SS, Chaerkady R, et al (2014) A draft map of the human proteome. Nature 509: 575–581. 10.1038/nature13302 24870542 PMC4403737

[15] Wilhelm M, Schlegl J, Hahne H, Moghaddas GA, Lieberenz M, Savitski MM, et al (2014) Mass-spectrometry-based draft of the human proteome. Nature 509: 582–587. 10.1038/nature13319 24870543

[16] Shuainan Liu QLSS (2012) The application of 2-NBDG as a fluorescent tracer for assessing hepatic glucose production in mice during hyperinsulinemic euglycemic clamp. Acta Pharmaceutica Sinica B. pp. 403–410.

[17] Ho JH, Lee OK, Fu YJ, Shih HT, Tseng CY, Chung CC, et al (2013) An iTRAQ proteomic study reveals an association between diet-induced enhanced fatty acid metabolism and the development of glucose intolerance in prediabetic mice. J Proteome Res 12: 1120–1133. 10.1021/pr300662j 23316967

[18] Susin SA, Lorenzo HK, Zamzami N, Marzo I, Snow BE, Brothers GM, et al (1999) Molecular characterization of mitochondrial apoptosis-inducing factor. Nature 397: 441–446. 9989411 10.1038/17135

[19] Shoshan-Barmatz V, Gincel D (2003) The voltage-dependent anion channel: characterization, modulation, and role in mitochondrial function in cell life and death. Cell Biochem Biophys 39: 279–292. 14716081 10.1385/CBB:39:3:279

[20] Trempe JF, Fon EA (2013) Structure and Function of Parkin, PINK1, and DJ-1, the Three Musketeers of Neuroprotection. Front Neurol 4: 38 10.3389/fneur.2013.00038 23626584 PMC3630392

[21] Poynton RA, Hampton MB (2014) Peroxiredoxins as biomarkers of oxidative stress. Biochim Biophys Acta 1840: 906–912. 10.1016/j.bbagen.2013.08.001 23939310

[22] Bonnefont JP, Djouadi F, Prip-Buus C, Gobin S, Munnich A, Bastin J (2004) Carnitine palmitoyltransferases 1 and 2: biochemical, molecular and medical aspects. Mol Aspects Med 25: 495–520. 15363638 10.1016/j.mam.2004.06.004

[23] Eaton S, Bartlett K, Quant PA (2001) Carnitine palmitoyl transferase I and the control of beta-oxidation in heart mitochondria. Biochem Biophys Res Commun 285: 537–539. 11444876 10.1006/bbrc.2001.5201

[24] Pintus F, Floris G, Rufini A (2012) Nutrient availability links mitochondria, apoptosis, and obesity. Aging (Albany NY) 4: 734–741. 23211444 10.18632/aging.100505 PMC3560440

[25] Parysow O, Mollerach AM, Jager V, Racioppi S, San RJ, Gerbaudo VH (2007) Low-dose oral propranolol could reduce brown adipose tissue F-18 FDG uptake in patients undergoing PET scans. Clin Nucl Med 32: 351–357. 17452860 10.1097/01.rlu.0000259570.69163.04

[26] Skarulis MC, Celi FS, Mueller E, Zemskova M, Malek R, Hugendubler L, et al (2010) Thyroid hormone induced brown adipose tissue and amelioration of diabetes in a patient with extreme insulin resistance. J Clin Endocrinol Metab 95: 256–262. 10.1210/jc.2009-0543 19897683 PMC2805496

[27] Fromme T, Klingenspor M (2011) Uncoupling protein 1 expression and high-fat diets. Am J Physiol Regul Integr Comp Physiol 300: R1–R8. 10.1152/ajpregu.00411.2010 21048077

[28] Vincent HK, Taylor AG (2006) Biomarkers and potential mechanisms of obesity-induced oxidant stress in humans. Int J Obes (Lond) 30: 400–418. 16302012 10.1038/sj.ijo.0803177

[29] Vasagiri N, Kutala VK (2014) Structure, function, and epigenetic regulation of BNIP3: a pathophysiological relevance. Mol Biol Rep. 10.1007/s11033-014-3664-x 25096512

[30] Op DBK, Schacht J, Van Camp G (2011) Apoptosis in acquired and genetic hearing impairment: the programmed death of the hair cell. Hear Res 281: 18–27. 10.1016/j.heares.2011.07.002 21782914 PMC3341727

[31] Lin MT, Beal MF (2006) Mitochondrial dysfunction and oxidative stress in neurodegenerative diseases. Nature 443: 787–795. 17051205 10.1038/nature05292

[32] Odorizzi G, Babst M, Emr SD (2000) Phosphoinositide signaling and the regulation of membrane trafficking in yeast. Trends Biochem Sci 25: 229–235. 10782093 10.1016/s0968-0004(00)01543-7

[33] Miramar MD, Costantini P, Ravagnan L, Saraiva LM, Haouzi D, Brothers G, et al (2001) NADH oxidase activity of mitochondrial apoptosis-inducing factor. J Biol Chem 276: 16391–16398. 11278689 10.1074/jbc.M010498200

[34] Daugas E, Susin SA, Zamzami N, Ferri KF, Irinopoulou T, Larochette N, et al (2000) Mitochondrio-nuclear translocation of AIF in apoptosis and necrosis. FASEB J 14: 729–739. 10744629

[35] Joza N, Susin SA, Daugas E, Stanford WL, Cho SK, Li CY, et al (2001) Essential role of the mitochondrial apoptosis-inducing factor in programmed cell death. Nature 410: 549–554. 11279485 10.1038/35069004

[36] Loeffler M, Daugas E, Susin SA, Zamzami N, Metivier D, Nieminen AL, et al (2001) Dominant cell death induction by extramitochondrially targeted apoptosis-inducing factor. FASEB J 15: 758–767. 11259394 10.1096/fj.00-0388com

[37] Cregan SP, Fortin A, MacLaurin JG, Callaghan SM, Cecconi F, Yu SW, et al (2002) Apoptosis-inducing factor is involved in the regulation of caspase-independent neuronal cell death. J Cell Biol 158: 507–517. 12147675 10.1083/jcb.200202130 PMC2173837

[38] Zhang X, Chen J, Graham SH, Du L, Kochanek PM, Draviam R, et al (2002) Intranuclear localization of apoptosis-inducing factor (AIF) and large scale DNA fragmentation after traumatic brain injury in rats and in neuronal cultures exposed to peroxynitrite. J Neurochem 82: 181–191. 12091479 10.1046/j.1471-4159.2002.00975.x

[39] De Pinto V, Reina S, Guarino F, Messina A (2008) Structure of the voltage dependent anion channel: state of the art. J Bioenerg Biomembr 40: 139–147. 10.1007/s10863-008-9140-3 18668358

[40] Ben-Hail D, Palty R, Shoshan-Barmatz V (2014) Measurement of mitochondrial Ca2+ transport mediated by three transport proteins: VDAC1, the Na+/Ca2+ exchanger, and the Ca2+ uniporter. Cold Spring Harb Protoc 2014: 161–166. 10.1101/pdb.top066241 24492769

[41] Shimizu S, Ide T, Yanagida T, Tsujimoto Y (2000) Electrophysiological study of a novel large pore formed by Bax and the voltage-dependent anion channel that is permeable to cytochrome c. J Biol Chem 275: 12321–12325. 10766872 10.1074/jbc.275.16.12321

[42] Adachi M, Higuchi H, Miura S, Azuma T, Inokuchi S, Saito H, et al (2004) Bax interacts with the voltage-dependent anion channel and mediates ethanol-induced apoptosis in rat hepatocytes. Am J Physiol Gastrointest Liver Physiol 287: G695–G705. 15044178 10.1152/ajpgi.00415.2003

[43] Shimizu S, Narita M, Tsujimoto Y (1999) Bcl-2 family proteins regulate the release of apoptogenic cytochrome c by the mitochondrial channel VDAC. Nature 399: 483–487. 10365962 10.1038/20959

[44] Tsujimoto Y, Shimizu S (2002) The voltage-dependent anion channel: an essential player in apoptosis. Biochimie 84: 187–193. 12022949 10.1016/s0300-9084(02)01370-6

[45] Shoshan-Barmatz V, Keinan N, Abu-Hamad S, Tyomkin D, Aram L (2010) Apoptosis is regulated by the VDAC1 N-terminal region and by VDAC oligomerization: release of cytochrome c, AIF and Smac/Diablo. Biochim Biophys Acta 1797: 1281–1291. 10.1016/j.bbabio.2010.03.003 20214874

[46] Vande VC, Cizeau J, Dubik D, Alimonti J, Brown T, Israels S, et al (2000) BNIP3 and genetic control of necrosis-like cell death through the mitochondrial permeability transition pore. Mol Cell Biol 20: 5454–5468. 10891486 10.1128/mcb.20.15.5454-5468.2000 PMC85997

[47] Vasagiri N, Kutala VK (2014) Structure, function, and epigenetic regulation of BNIP3: a pathophysiological relevance. Mol Biol Rep. 10.1007/s11033-014-3664-x 25096512

[48] Joshi A, Bondada V, Geddes JW (2009) Mitochondrial micro-calpain is not involved in the processing of apoptosis-inducing factor. Exp Neurol 218: 221–227. 10.1016/j.expneurol.2009.04.013 19393648 PMC2756010

[49] Yang Y, Smith DJ, Keating KD, Allison DB, Nagy TR (2014) Variations in body weight, food intake and body composition after long-term high-fat diet feeding in C57BL/6J mice. Obesity (Silver Spring) 22: 2147–2155. 10.1002/oby.20811 24942674 PMC4180788

