# Supplementary figures and images for: Comparative Proteome Analysis of Brown Adipose Tissue in Obese C57BL/6J Mice Using iTRAQ-Coupled 2D LC-MS/MS
Source: PLoS One. 2015 Mar 6;10(3):e0119350. doi: 10.1371/journal.pone.0119350 (PMC4352050; doi:10.1371/journal.pone.0119350)

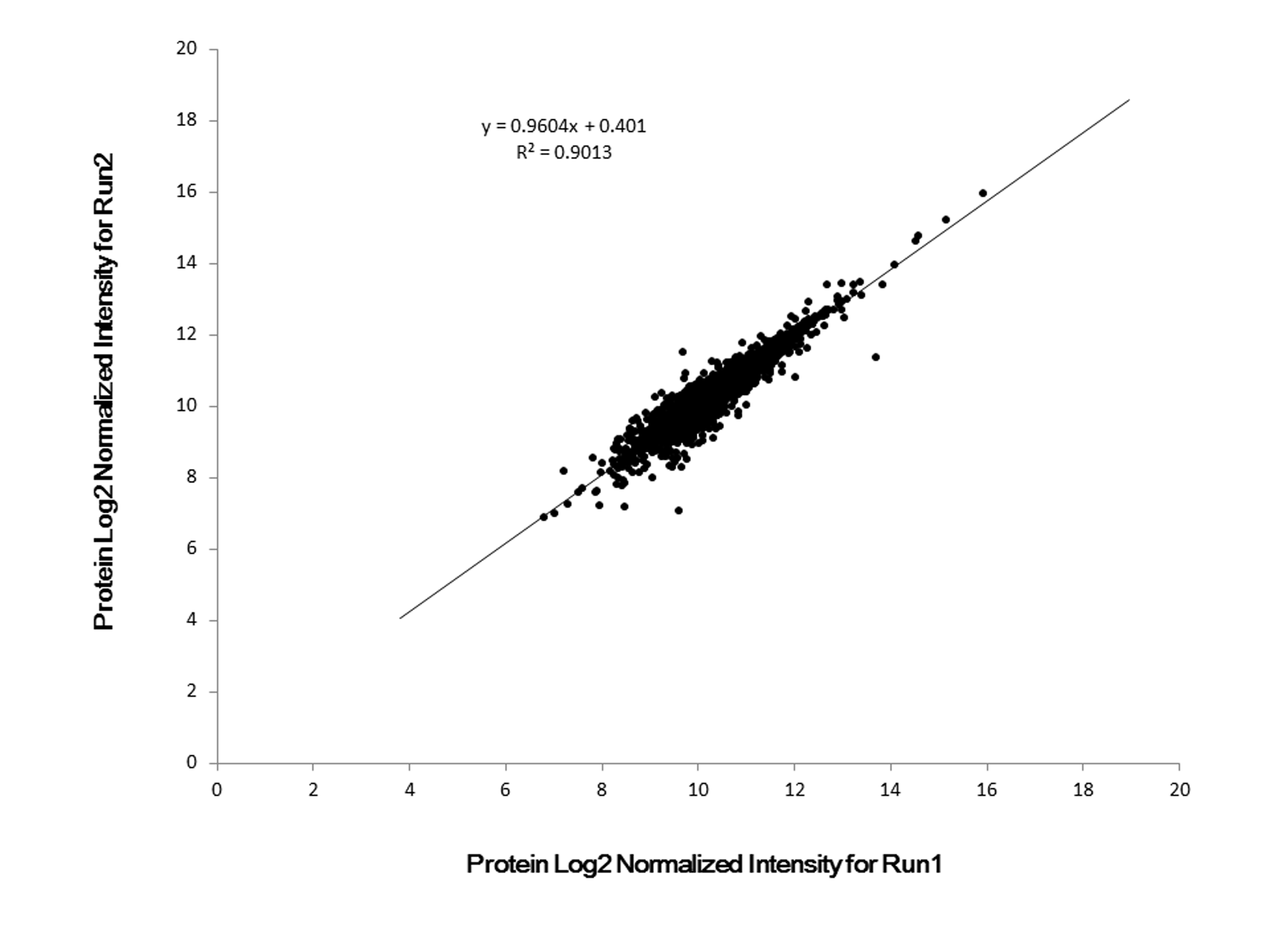

Supplement: S1 Fig — To evaluate the repeatability of experiments with LC-MS/MS, the correlations of normalized intensity of quantitative proteins between two runs were calculated. (TIF) [file pone.0119350.s001.tif]

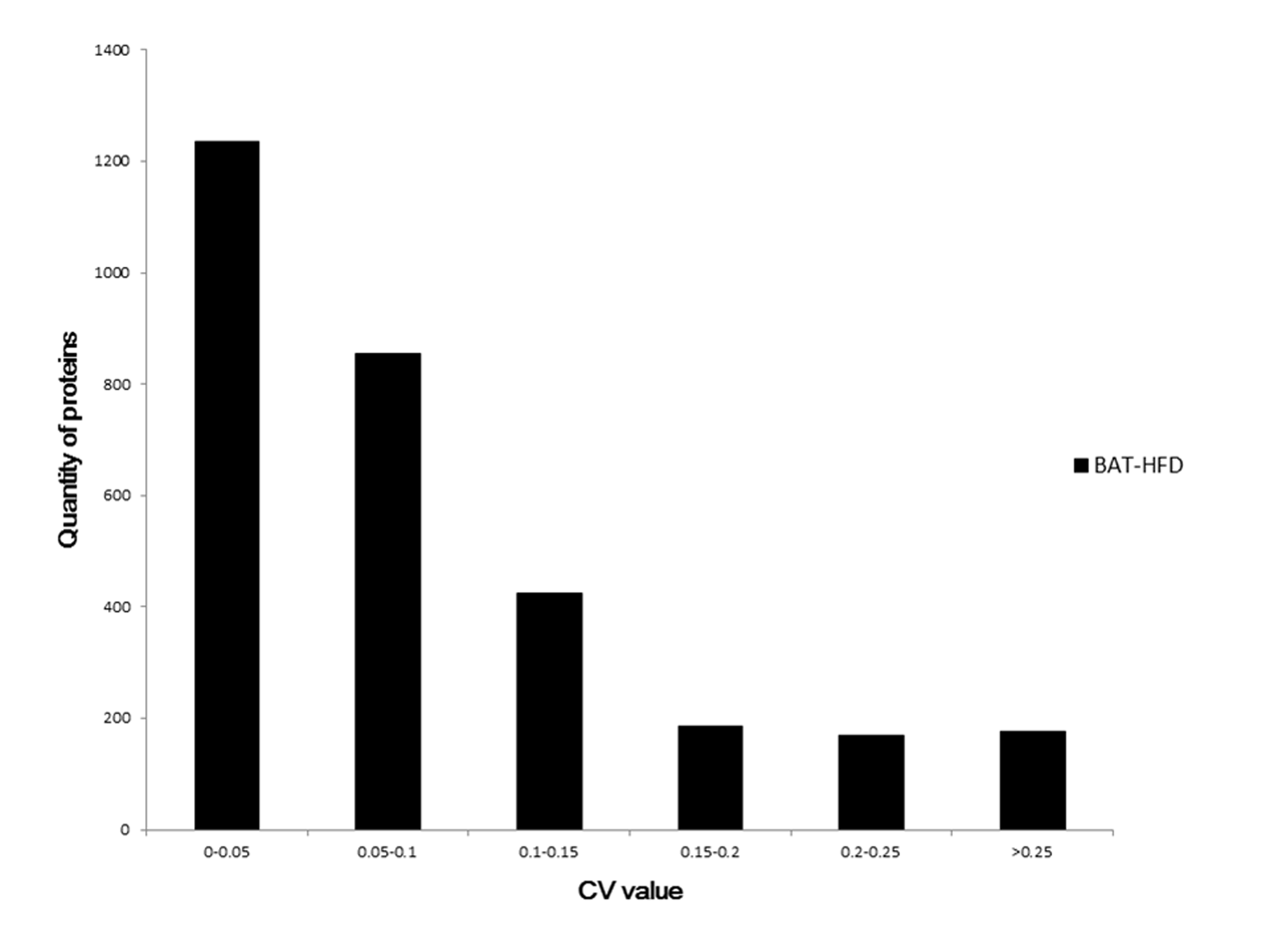

Supplement: S2 Fig — In order to minish technical error, proteins whose CV value greater than 0.2 were excluded. (TIF) [file pone.0119350.s002.tif]

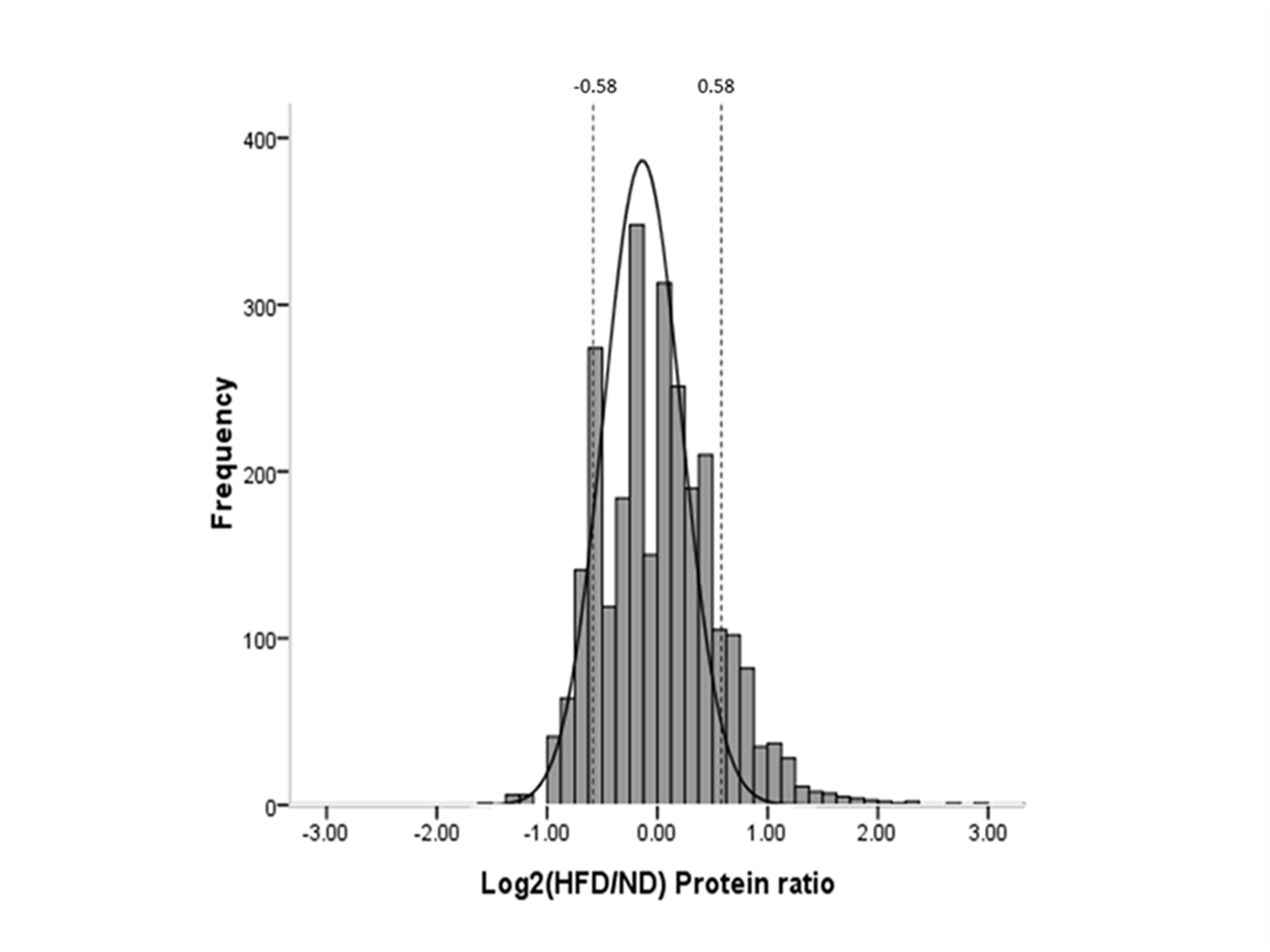

Supplement: S3 Fig — Fold change greater than 1.5 was considered as a threshold to minimize biological and technical errors. (TIF) [file pone.0119350.s003.tif]
